# Association of clozapine treatment and rate of methamphetamine or amphetamine relapses and abstinence among individuals with concurrent schizophrenia spectrum and amphetamine use disorder: A retrospective cohort study

**DOI:** 10.1177/02698811231191781

**Published:** 2023-08-04

**Authors:** Reza Rafizadeh, Laura Frankow, Hajer Mahmood, Sukhpreet Poonia, Nickie Mathew, Marlon Danilewitz, Chad A Bousman, William G Honer, Christian G Schütz

**Affiliations:** 1 Department of Experimental Medicine, University of British Columbia, Vancouver, BC, Canada; 2 Department of Psychiatry, University of British Columbia, Vancouver, BC, Canada; 3 Faculty of Pharmaceutical Sciences, University of British Columbia, Vancouver, BC, Canada; 4 BC Mental Health and Substance Use Services Research Institute, Vancouver, BC, Canada; 5 Red Fish Healing Centre for Mental Health & Addiction, Coquitlam, BC, Canada; 6 Lower Mainland Pharmacy Services, Vancouver, BC, Canada; 7 Departments of Psychiatry and Community Health Sciences, University of Calgary, Calgary, AB, Canada; 8 Mathison Centre for Mental Health Research & Education, Hotchkiss Brain Institute, University of Calgary, Calgary, AB, Canada; 9 Alberta Children’s Hospital Research Institute, University of Calgary, Calgary, AB, Canada; 10 Department of Psychiatry, University of Toronto, Toronto, ON, Canada

**Keywords:** Methamphetamine, clozapine, schizophrenia, substance use disorders, concurrent disorders, dual diagnosis

## Abstract

**Background:**

Preliminary evidence suggest clozapine is associated with more favorable impact on concurrent substance use disorder related outcomes in patients with concurrent schizophrenia spectrum disorders (SSD). At the same time, there is a dearth of evidence with regards to clozapine outcomes in the context of concurrent methamphetamine or amphetamine use disorder (MAUD).

**Aims:**

To examine whether clozapine use decreases rate of methamphetamine or amphetamine (MA) relapses and increases the likelihood of maintaining abstinence from any MA use.

**Methods:**

A descriptive-analytic retrospective cohort study was conducted on individuals with SSD-MAUD in an inpatient provincial treatment and rehabilitation center for concurrent disorders. Antipsychotic exposure was categorized as “on clozapine” or “on other antipsychotic(s).” Data were collected using electronic health records. Logistic regression was used to examine association of clozapine treatment with likelihood of complete abstinence from MA use for the duration of antipsychotic exposure. Negative binomial regression was used to examine association of clozapine treatment with rate of MA relapses for the duration of antipsychotic exposure.

**Results:**

The majority of the 87 included patients were male. Ethnicity was diverse, with the largest groups self-identifying as Indigenous and European. Clozapine use was both associated with increased likelihood of maintaining abstinence from MA use (adjusted odds ratio (aOR) = 3.05, 95% confidence intervals (CI) = 1.15–8.1, *p* = 0.025), and decreased rate of MA relapses (aRR = 0.45, 95% CI = 0.25–0.82, *p* = 0.009) for the duration of antipsychotic exposure. Co-prescription of psychostimulants was associated with increased rate of MA relapses (aRR = 2.43, 95% CI = 1.16–5.10, *p* = 0.019).

**Conclusion(s):**

In this study, clozapine use compared with other antipsychotics in SSD was associated with improved outcomes related to severe concurrent MAUD. Co-prescription of psychostimulant medications was associated with a poor outcome.

## Background

Methamphetamine or amphetamine use disorder (MAUD) has continued to increase in prevalence across North America, leading to significant public health concerns (Casey, 2019; Glasner-Edwards and Mooney, 2014; Jones et al., 2020). Methamphetamine (MA)-related hospitalizations have tripled from 55,447 to 206,180 hospitalizations in the United States between 2008 and 2015 (Winkelman et al., 2018); and MA-related emergency department (ED) visits have increased by 15 folds from 2003 to 2020 in the most populated province in Canada (Crispo et al., 2023). It is noteworthy that re-visiting the ED for any reason was independently associated with a diagnosis of psychosis, aOR = 1.54, 95% CI = 1.30–1.83 (Crispo et al., 2023); signifying that it appears essential to refer these individuals to specialty care services for treatment of concurrent disorders in order to reduce avoidable ED visits and associated cost and resource utilization (Crispo et al., 2023).

Globally, there is a high prevalence of substance use disorders (SUD) in patients with schizophrenia spectrum disorders (SSD) (Hunt et al. 2018). In the context of the increasing accessibility and availability at lower cost (Casey, 2019; Glasner-Edwards and Mooney, 2014; Jones et al., 2020), the risk of exposure to MA in individuals with SSD is high. Moreover, the relationship between SSD and MAUD is bidirectional: SSD is a risk factor for developing MAUD; and MAUD is a risk factor for precipitating intermittent or prolonged psychosis (Bousman et al., 2011; Glasner-Edwards and Mooney, 2014) with a subpopulation at risk of conversion to a chronic and persistent psychotic disorder (Akiyama et al., 2011; Starzer et al., 2018). Additionally, the SSD population may be more susceptible to developing antipsychotic-nonresponsive SSD due to MA use and common neurological comorbidities like head injury, learning disability, and attention deficit hyperactivity disorder (ADHD) (Fujii, 2002); and this may implicate the need for clozapine treatment.

Antipsychotic medications are the mainstay of treatment in SSD; yet, there is sparse research examining their impact on highly prevalent concurrent SUD outcomes. Some preliminary evidence, mainly from observational studies, have shown that clozapine is associated with more favorable effects on improving concurrent SUD outcomes compared with other antipsychotic medications (Rafizadeh et al., 2022). There is, however, a dearth of evidence about its effects on outcomes related to concurrent MAUD in particular.

The primary goal of this study was to compare the effects of clozapine treatment with other antipsychotic medications on MAUD-related outcomes such as abstinence and rate of substance use relapse in a well-characterized SSD population, who had a concurrent DSM 5 diagnosis of severe MAUD requiring inpatient treatment for concurrent disorders due to ongoing MA use relapse resulting in psychotic decompensation.

## Materials and methods

### Study setting and population

This was a retrospective cohort study using routine care clinical data of patients admitted to the Burnaby Center for Mental Health and Addiction (BCMHA) in British Columbia, Canada (renamed in late 2021 to Red Fish Healing Center for Mental Health & Addiction, when it was relocated to Coquitlam, British Columbia). Ethics approval was obtained from the University of British Columbia Clinical Research Ethics Board as well as the BC Mental Health and Substance Use Services ethics committee, and employed principles highlighted in the Declaration of Helsinki.

The BCMHA was a 94-bed provincial tertiary treatment facility with an average length of stay of 20 weeks that provided multidisciplinary integrated treatment for adults (19 years of age or older) with concurrent SUDs and severe and persistent mental illnesses. According to the access protocol, the patients must have failed other programs on a regional level and must have significant issues in each of the four identified domains: mental health, substance use, physical health, and behavioral. Furthermore, clients eligible for admission must have been unable to adequately engage with, receive services from, or benefit from traditional mental health and addiction programs. Reported changes in the patient population at BCMHA, based on a comparison of cross-sectional interviews conducted in 2009/2010 and again from 2018 until 2020, shows that the most frequently used substance before intake has become MA (63.7%). Consistent with the House of Commons Standing Committee on Health’s report (Casey, 2019), MA has shown the greatest increase among all substances in this population with concomitant increase in diagnosis of psychotic disorders. There is existing literature describing characteristics of admitted patients to BCMHA (Lee-Cheong et al., 2021; Schütz et al., 2013).

Admitted patients were qualified for independent passes as they moved along in the treatment plan and upon confirmed negative breathalyzer test and immunoassay point of care urine drug screen (UDS) results. Confirmatory testing with mass spectrometry at a provincial laboratory was performed in cases of suspicions for false positive immunoassay results. Upon return from passes, patients were assessed clinically by a nurse. Nurses were required to perform a mental status exam to identify complications such as psychosis, cognitive impairments, and risk of harm to self or others; and any deviation from baseline raised suspicions of substance use relapse with requirement for breathalyzer and UDS. Concurrently, UDS were performed on an ad hoc random basis minimally twice per month. Thereafter, for every proven positive UDS result, passes were withheld until a negative UDS was obtained and documented prior to re-initiating passes. Consequently, a positive UDS result or breathalyzer test following issuance of a pass was considered a relapse to substance use. Patients who had positive UDS findings for prescription drugs were counted as having negative results if their mental status was not assessed to have changed. For instance, positive UDS for amphetamine but not methamphetamine in patients who were administered lisdexamfetamine was counted as a negative result in the absence of changes in mental status. Accordingly, positive UDS results for both methamphetamine and amphetamine were counted as a relapse to MA use.

### Eligibility criteria

Electronic charts of patients with DSM 5 diagnosis of SSD was reviewed for inclusion over the period of December 8, 2019 to October 8, 2021. The date range was chosen as electronic charts were not available prior to December 8, 2019 and the end-date was the date that this facility moved to a new location for further expansion of services. Patients were included if they (1) had stayed at the center for a minimum of 90 days during the study period (the 90 days was considered the minimum required stay to finish treatment at the facility); (2) had a concurrent DSM 5 diagnosis of severe MAUD (MA had to be the substance of choice in poly-drug users and primary drug of concern resulting in ongoing psychotic decompensation requiring inpatient treatment at BCMHA); (3) had at least two previous antipsychotic medication trials with adequate dose/duration without significant clinical improvement; and consequently, were eligible for clozapine treatment.

### Independent exposure groups and the duration of observation

Exposure to medication was categorized as “on clozapine” or “on other antipsychotic(s).” The patient had to have been be taking clozapine for at least 90 days to be considered for the “on clozapine.” Ninety days was chosen based on the criteria for an “adequate” clozapine trial (Howes et al., 2017). If patients were taking multiple antipsychotic medications, and one of these was clozapine, the patient would be categorized as “on clozapine.” The duration of observation was based on antipsychotic exposure periods; that is the days on clozapine in the “on clozapine” group or days on other antipsychotics in “other antipsychotic(s)” group. For patients who were started on clozapine after admission to BCMHA, the days prior to clozapine initiation were excluded from analysis. Within-individual analysis was not possible due to the fact that time to initiation of clozapine was short (see results). For patients who were on clozapine but did not meet the 90 days criteria, if they were still on other antipsychotics for 90 days, they were included in the analysis as “on other antipsychotic(s).” The days that they were on clozapine were excluded from analysis. All included patients were observed until the point of discharge from the facility.

### Data collection

Included patients were anonymized by medical record number with a study ID and a chart review was conducted via electronic medical records. Documents reviewed included referral packages, clinical notes, patient records, lab results (including UDS and confirmatory testing to rule out false positives), consultations, admission and discharge documentation, and medication administration records. Data extracted by two authors (H.M. and L.F.) utilizing a custom data extraction template. It included patient demographics, medical and psychiatric diagnoses, detailed substance use history (substances used, severity, route, and date of last use prior to admission), the Health of the Nation Outcome Scales (HONOS) scores (a clinician rated instrument comprising scales measuring domains such as cognition, hallucination and delusions, mania, depression and anxiety rated on a five-point item of severity of 0–4), prescribed medications, and relapse to substance use confirmed by positive UDS and breathalyzer results throughout their stay at the facility. The principal investigator (R.R.) oversaw consistency of retrospective data extraction. Data were extracted between June 2022 and August 2022.

### Outcomes

The main non-abstinence-based outcome measure was rate of MA relapses as validated by positive UDSs for the duration of antipsychotic exposure. Our abstinence-based outcome measure was maintaining abstinence from any MA use for the duration of antipsychotic exposure. Given the poly-drug use seen in our patient population, the secondary outcome measure was abstinence and rate of any substance use relapse (other than nicotine) for the duration of antipsychotic exposure.

### Covariates

Independent covariates included in our analysis were the duration of antipsychotic exposure, sex (male or female), age in increments of every 10 years, chlorpromazine (CPZ)-equivalent total antipsychotic daily dose in increments of every 100 mg, and co-prescribed medications (antidepressants, mood stabilizers, psychostimulant medications, long-acting injectable (LAI) antipsychotics, SUD pharmacotherapy, and opioid agonist therapy (OAT)). We used increments of 10 years for age and 100 mg for CPZ-equivalence to improve clinical interpretation of odds and risk ratios derived from our regression model. For the list of co-prescribed medications and their doses, refer to Supplemental Table 1. For the list of prescribed, antipsychotics refer to Supplemental Table 2.

Total maximum antipsychotic daily dose in both groups was determined by converting the total daily dose of all antipsychotics to CPZ equivalents using the American Association of Pharmacists’ Antipsychotic Dose Equivalents table (Patel et al., 2020)

### Statistical analysis

Given that substance use relapse is a recurrent event and a count variable, and that our data were overdispersed, that is when variance exceeds the mean, we used negative binomial regression in our analysis using IBM^®^ SPSS™ Statistics for Windows version 27 (IBM Corp, New York, NY, USA) and Microsoft Excel 365. Overdispersion is expected for contiguous events where the first occurrence makes a second occurrence more likely, independent from this still being random. Given that our mean was relatively low, negative binomial regression was deemed an appropriate model to deal with the potential of a large number of values being zero (Warton, 2005; Xie et al., 2013). Statistical significance was set to 0.05, and the results were reported as adjusted rate ratios (aRRs) with 95% CIs.

We used logistic regression for analysis of likelihood of maintaining abstinence for the duration of antipsychotic medication pharmacotherapy, and the results were reported as adjusted odds ratios (aORs).

### Sensitivity analysis

Given that antipsychotic polytherapy was highly prevalent in both exposure groups, to examine the robustness of the results, we performed a sensitivity analysis comparing clozapine monotherapy (*n* = 12) with “other antipsychotics” group (*n* = 48) in terms of association with rate of relapses to MA.

## Results

### Cohort characteristics

**Figure 1.**
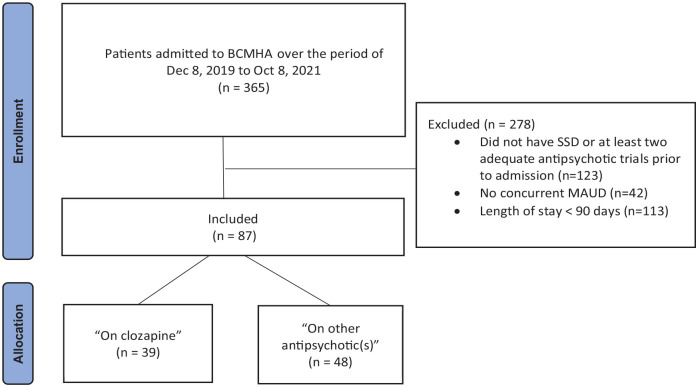
Flow chart of the patient selection.

Out of 365 patients admitted to the program during the study period, 87 patients met the inclusion criteria. Thirty-nine of these patients were taking clozapine for at least 90 days and were allocated to the “on clozapine” (Figure 1). Table 1 shows clinical and demographic characteristics of the study population. Approximately half of the population was diagnosed with schizophrenia and half with schizoaffective disorder. Most included patients were male and ancestry was diverse, with the two largest groups self-identifying as Indigenous and European. All included patients had either concurrent active, or in-early-remission in a controlled environment (as some were transferred from a locked facility) concurrent DSM 5 diagnosis of severe MAUD at the point of admission. MA was the drug of choice for all included patients and the primary drug of concern resulting in ongoing psychotic decompensation requiring inpatient treatment at BCMHA. The mean number of days since last use was 45 (range: 0–210). Smoking was the most common route of MA administration (49%), followed by intravenous (18%), snorting (15%), and unknown (18%). Seventeen patients (44%) in the “on clozapine” group had their clozapine initiated after admission to BCMHA. For these seventeen patients, the duration (days) of non-clozapine antipsychotic treatment prior to initiation of clozapine was 35.5 ± 21.0; and these days were excluded from our analysis. None of the patients in the “on clozapine” group discontinued their clozapine medication. One patient in “other antipsychotic(s)” group was on clozapine for the first 60 days of admission; however, clozapine was discontinued secondary to neutropenia. These 60 days were excluded from our analysis. All the included patients were receiving financial and health support under designation of Person with Disabilities under government of British Columbia.

**Table 1.** Clinical and demographic characteristics of the study population.

	All patients (*n* = 87)	“on clozapine” (*n* = 39)	“on other antipsychotic(s)” (*n* = 48)
Age, years, mean (SD)	33.5 ± 10.0	33.9 ± 9.7	33.2 ± 10.4
Sex, female, *n* (%)	31 (35.6%)	14 (35.9%)	17 (35.4%)
Ancestry, *n* (%)			
• Indigenous	36 (41.3%)	15 (38.5%)	21 (43.8%)
• European	25 (28.7%)	13 (33.3%)	12 (25.0%)
• East Asian	2 (2.3%)	0 (0.0%)	2 (4.2%)
• South Asian	4 (4.6%)	1 (2.6%)	3 (6.3%)
• African	2 (2.3%)	2 (5.1%)	0 (0.0%)
• Hispanic	1 (1.1%)	1 (2.6%)	0 (0.0%)
• Unspecified	17 (19.5%)	7 (17.9%)	10 (20.8%)
Weight			
• On admission, mean kg (SD)		82.0 ± 15.2	84.0 ± 24.0
• At the end of observation period, mean kg (SD)		89.1 ± 23.4	85.5 ± 18.4
Diagnosis			
• Schizophrenia, *n* (%)	45 (51.7%)	22 (56.4%)	23 (47.9%)
• Schizoaffective disorder, *n* (%)	42 (48.3%)	17 (43.6%)	25 (52.1%)
○ Duration of psychosis, mean years (SD)	9.0 ± 7.9	8.9 ± 6.8	9.1 ± 8.8
• MAUD, *n* (%)	87 (100%)	39 (100%)	48 (100%)
• OUD, *n* (%)	51 (59%)	21 (54%)	30 (62.5%)
• AUD, *n* (%)	49 (56%)	21 (51.3%)	28 (58.3 %)
• CUD, *n* (%)	77 (89%)	33 (84.6%)	34 (70.8 %)
• Nicotine use disorder	82 (94.3%)	37 (94.9%)	45 (93.7%)
Duration of observation as per antipsychotic medication use			
• Mean days (SD)		196 ± 73.2	165 ± 67.3
Total UDS performed per patient over the duration of observation			
• Mean (SD)		29.69 ± 14.74	32.98 ± 15.01
Antipsychotic dosing characteristics at the end of observation period			
• Daily clozapine dose, mean mg (SD)		286.2 ± 101.9	
• CPZ-equivalent daily dose of non-clozapine antipsychotics, mean mg (SD)		188.0 ± 181.0	432.1 ± 327.2
• CPZ-equivalent total daily antipsychotic dose, mean mg (SD)		474 ± 210.4	432.1 ± 327.2
Number of concomitant antipsychotic medications at the end of observation period^a^			
• 1 antipsychotic, *n* patients (%)	41 (47.1%)	11 (28.2%)	30 (62.5%)
• 2 antipsychotics, *n* patients (%)	41 (47.1%)	25 (64.1%)	16 (33.3%)
• 3 antipsychotics, *n* patients (%)	5 (5.7%)	3 (7.7%)	2 (4.2%)
• On LAI antipsychotics, *n* patients (%)	44 (50.6%)	12 (30.8%)	32 (66.7%)
Co-prescribed medications^a^			
• Mood stabilizer medication, *n* patients (%)	30 (34.5%)	17 (43.6%)	13 (27.1%)
• Antidepressant medication, *n* patients (%)	24 (27.6%)	11 (28.2%)	13 (27.1%)
• Psychostimulant medication, *n* patients (%)	13 (14.9%)	5 (12.8%)	8 (16.7%)
• SUD medication, *n* patients (%)	46 (53%)	21 (54%)	25 (52%)
• OAT, *n* patients (%)	32 (36.8%)	14 (35.9%)	18 (37.5%)
HONOS on admission^b^			
• Problems with cognition		2.07 ± 1.03	2.04 ± 0.79
• Problems with hallucinations or delusions		3.13 ± 0.64	2.89 ± 1.03
• Problems with depressed mood		1.93 ± 0.96	2.36 ± 1.10
• Problems with mania		1.13 ± 1.30	0.89 ± 1.26
• Problems with anxiety		3.0 ± 0.38	2.61 ± 0.92
HONOS at the end of observation period^c^			
• Problems with cognition		0.9 ± 1.03	0.59 ± 0.94
• Problems with hallucinations or delusions		1.23 ± 1.25	0.78 ± 0.97
• Problems with depressed mood		0.23 ± 0.62	0.23 ± 0.53
• Problems with mania		0.03 ± 0.18	0.03 ± 0.16
• Problems with anxiety		0.4 ± 0.81	0.15 ± 0.36

AUD: alcohol use disorder; CUD: cannabis use disorder; LAI: long acting injectable; MAUD: methamphetamine or amphetamine use disorder; OAT: opioid agonist therapy = buprenorphine, methadone, and long acting morphine; OUD: opioid use disorder.

a Types and doses of antipsychotic and co-prescribed medications used is available in Supplemental Tables S1 and S2.

b For admission, 24 patients in the “on clozapine” group were missing HONOS scores; 20 patients in the “other antipsychotic(s)” group were missing HONOS scores.

c At the end of observation period, nine patients in the “on clozapine” group were missing HONOS scores; nine patients in the “other antipsychotic(s)” group were missing HONOS scores.

### Outcomes

#### Likelihood of maintaining abstinence

For the duration of observation, 51 (59%) patients had at least one MA relapse. Clozapine treatment was associated with increased likelihood of maintaining abstinence from any MA use (aOR = 3.05, 95% CI = 1.15–8.1, *p* = 0.025).

For the duration of observation, 65 (75%) patients had at least one relapse to any substance use (other than nicotine). Clozapine treatment was associated with increased likelihood of maintaining abstinence from any substance use (aOR = 4.76, 95% CI = 1.29–17.8, *p* = 0.02).

#### Rate of substance use relapses

**Figure 2.**
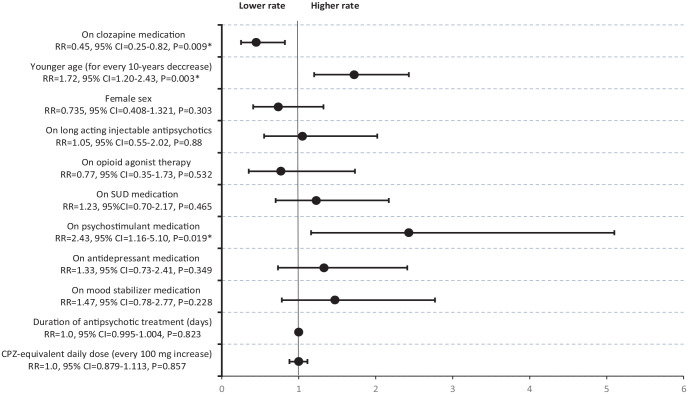
Adjusted rate ratios and 95% CI for the rate of methamphetamine or amphetamine relapses in inpatients with schizophrenia spectrum and amphetamine use disorder.

For the duration of observation, clozapine treatment was associated with decreased rate of MA relapses (aRR = 0.45, 95% CI = 0.25–0.82, *p* = 0.009). Co-prescription of psychostimulant medications (aRR = 2.43, 95% CI = 1.16–5.10, *p* = 0.019) and younger age (aRR = 1.72, 95% CI = 1.20–2.43, *p* = 0.003), measured in 10-years-increments, were associated with increased rate of MA relapses (Figure 2).

For the duration of observation, clozapine treatment was associated with decreased rate of any substance use relapses (other than nicotine) (aRR = 0.36, 95% CI = 0.22–0.60, *p* < 0.001).

#### Sensitivity analysis

Clozapine monotherapy treatment (*n* = 12) in comparison with other antipsychotic medications (*n* = 48) was associated with decreased rate of MA relapses (aRR = 0.34, 95% CI = 0.24–0.49, *p* = 0.043); clozapine monotherapy treatment did not have statistically significant association with likelihood of maintaining abstinence (aOR = 2.22, 95% CI = 0.43–6.67, *p* = 0.45).

## Discussion

In the face of the growing public health crisis related to MA, our study provides insights on best practices for management of SSD-MAUD. This population, while a subset of the population of those with MAUD, represents a high service need group, with limited available treatment options. Our study highlights that compared with other antipsychotic medications, clozapine treatment was associated with both statistically significant decreased rate of MA relapses and increased likelihood of maintaining abstinence. Moreover, this association remained statistically significant for rate of any substance use relapses (nicotine was not assessed). These findings are kept with previously reported findings suggesting favorable impact of clozapine in SSD-SUD (Rafizadeh et al., 2022).

In our study, we observed similar clinician ratings in domains of psychosis, mood, cognition, and anxiety at the end of observation period in both antipsychotic exposure groups. Although we had no information on the temporality and severity of psychotic symptoms at different time points during their admission, this observation suggests that the association between clozapine and improved MAUD-related outcomes may go beyond clozapine’s attributed superior efficacy for both positive and negative symptoms of SSD (Wagner et al., 2021). Clozapine’s unique modulation of several neurotransmitter systems may independently impact rate of MA relapses and likelihood of abstinence.

The neurobiological characteristics of prolonged exposure to MA use, such as striatal dopamine downregulation (Ashok et al., 2017), persistently high glutamate content in anterior cingulate cortex, and deficits in γ-aminobutyric acid (GABA) inhibitory neurotransmission (Hsieh et al., 2014), may suggest that clozapine is a promising treatment option. Clozapine has selective binding and occupies relatively low levels of dopamine type-2 receptors in striatum and does not contribute to further striatal dopaminergic downregulation (Pilowski and Mulligan, 1997). Additionally, Clozapine’s propensity to increase GABA-B-mediated inhibitory neurotransmission (Nair et al., 2020) and to decrease glutamate levels (McQueen et al., 2021) may be of significance in reducing the extent of craving and attenuating MA reward effects.

In this study, clozapine use did not result in reduction in antipsychotic polypharmacy as suggested by previous published literature (Chong et al., 2000; Ochi et al., 2022); and a considerable amount of patients remained on concurrent LAI antipsychotics. Despite this, overall antipsychotic daily dose was similar between both exposure groups, and clozapine treatment did not push patients into the high-daily-dose range. This may suggest that the clinicians are reluctant to discontinue concomitant LAI antipsychotics in this population secondary to fear of non-adherence to clozapine post-discharge. Moreover, some preliminary evidence in SSD-SUD population suggests combination of clozapine, and an LAI antipsychotic may be associated with decrease in risk of ED visits and hospitalization in outpatient community settings (Grimminck et al., 2020)

Regarding the impact of additional factors on the MAUD outcomes, both younger age (measured in 10 years increments) and co-prescribed psychostimulant medications were associated with increased rate of MA relapses. Younger age has been associated with increase in substance use in previously published literature (Substance Abuse and Mental Health Services Administration, 2021). The role of psychostimulant pharmacotherapy in the treatment of MAUD remains unclear and controversial. While evidence supporting the benefits of substitution therapy in MAUD is emerging (Heikkinen et al., 2022; Tardelli et al., 2020), this evidence has been specific to patients without concurrent SSD or other persistent and severe mental illnesses. Ultimately, further research with regard to benefits and limitations of psychostimulants in the treatment of stimulant use disorders or ADHD, with and without concurrent SSD, is gravely needed.

Given the possibility that pharmacotherapy for an SUD may also improve outcomes related to other SUDs in poly-drug users, we included co-prescribed SUD medications and OAT as independent covariates in our analysis of rate of MA use relapses and abstinence; however, they had no association. Similarly, co-prescribing antidepressants and mood stabilizers had no association with abstinence or rate of MA use relapses in this population.

### Limitations

First, prescribing in our study was based on clinical judgment rather than randomized assignment, and generally clozapine treatment is selected for patients who are harder to treat and are not successfully treated with non-clozapine antipsychotics. Furthermore, prescribers may be inclined to prescribe clozapine to patients who they perceive as more adherent to oral medications post-discharge; consequently, caution should be exercised in causal inferences. Second, given that antipsychotic polytherapy was highly prevalent in both exposure groups, it is difficult to attribute the cause to a single antipsychotic medication. Thus, we performed a sensitivity analysis exploring association of clozapine monotherapy with rate of MA relapses and likelihood of maintaining abstinence; and overall, the sensitivity analysis showed statistically significant association with adjusted rate of relapses and trended toward significance in terms of likelihood of maintaining abstinence. Furthermore, we used treatment with LAI antipsychotics as a covariate in our regression models, and they did not have statistically significant association with rates of MA relapse or maintaining abstinence. Despite this, prospective randomized studies are required to confirm this association for causal inferences. Third, given the evidence around rapid onset psychosis following abrupt withdrawal from clozapine (Moncrieff, 2006), our findings in a controlled inpatient setting with excellent medication adherence may not be generalizable to outpatient settings with high levels of non-adherence. Fourth, we were unable to learn about the variation in the extent to which the included patients used BCMHA’s available evidence-based psychosocial interventions for substance use relapse prevention, such as the Matrix Model and Contingency Management. Fifth, we had no information on the temporality and severity of withdrawal symptoms and cravings among included patients. Consequently, we evaluated favorable MAUD outcomes based on the rate of MA relapses and maintaining abstinence. Sixth, the study population may also have not been homogenous with several environmental factors like routines, outside connections, and the availability of MA. Last, given the retrospective nature of the study and lack of performed UDS upon return from each independent pass, it is possible that substance use relapse was missed. Furthermore, the possibility of false negatives in routine ad hoc random UDS results and the short half-life of MA may have impacted the robustness of the results. Also, as needed UDS performed by trained nurses introduces an element of rater bias. However, it is unlikely that this bias would affect the UDSs of one treatment group over another as the both exposure groups were tested under these conditions for the duration of observation and the testing was independent of group assignment.

Another limitation of this study is that we did not know whether co-prescription of psychostimulant medications was to treat ADHD or (off-label) MAUD due to lack of documentation by clinicians. Given the protopathic bias with regard to initiation of psychostimulant pharmacotherapy in patients most likely to relapse and small number of patients receiving psychostimulant pharmacotherapy in this study, our results may underestimate the putative beneficial effect.

## Conclusions

In summary, clozapine treatment compared with other antipsychotic medications was associated with a decreased rate of MA relapses and a higher likelihood of maintaining abstinence in a sample of inpatients with concurrent SSD-MAUD. If further evidence supports superiority of clozapine in outcomes related to severe MAUD in SSD population, clozapine initiation should be considered sooner along the treatment trajectory. However, caution in co-prescribing psychostimulant medications in SSD-MAUD is warranted until further research can establish that the benefits outweigh the risks.

## Supplemental Material

sj-docx-1-jop-10.1177_02698811231191781 – Supplemental material for Association of clozapine treatment and rate of methamphetamine or amphetamine relapses and abstinence among individuals with concurrent schizophrenia spectrum and amphetamine use disorder: A retrospective cohort study Click here for additional data file. Supplemental material, sj-docx-1-jop-10.1177_02698811231191781 for Association of clozapine treatment and rate of methamphetamine or amphetamine relapses and abstinence among individuals with concurrent schizophrenia spectrum and amphetamine use disorder: A retrospective cohort study by Reza Rafizadeh, Laura Frankow, Hajer Mahmood, Sukhpreet Poonia, Nickie Mathew, Marlon Danilewitz, Chad A Bousman, William G Honer and Christian G Schütz in Journal of Psychopharmacology
