# Supplementary material for: Association of clozapine treatment and rate of methamphetamine or amphetamine relapses and abstinence among individuals with concurrent schizophrenia spectrum and amphetamine use disorder: A retrospective cohort study
Source: J Psychopharmacol. 2023 Aug 4;37(10):1040–8. doi: 10.1177/02698811231191781 (PMC10612371; doi:10.1177/02698811231191781)
Supplement: sj-docx-1-jop-10.1177_02698811231191781 – Supplemental material for Association of clozapine treatment and rate of methamphetamine or amphetamine relapses and abstinence among individuals with concurrent schizophrenia spectrum and amphetamine use disorder: A retrospective cohort study [file sj-docx-1-jop-10.1177_02698811231191781.docx]

**Supplementary Table 1**

Description of co-prescribed medications

| **N = 87** | **39 (on clozapine)** | **48 (other antipsychotic(s))** |
| --- | --- | --- |
| **Psychostimulant medications** | n (mean daily dose, range) | n (mean daily dose, range) |
| *Methylphenidate* | 4 (50mg, 20-72) | 7 (47.14, 20-72) |
| *Lisdexamphetamine* | 1 (20mg) | 1 (60mg) |
| **SUD-medications** | n | n |
| *Acamprosate* | 1 | 1 |
| *Acamprosate + gabapentin* | - | 1 |
| *Naltrexone* | - | 2 |
| *Naltrexone + gabapentin* | 3 | - |
| *Gabapentin* | 1 | 3 |
| *Buprenorphine ^a^* | 11 | 14 |
| *Methadone ^a^* | 2 | 3 |
| *Morphine long acting ^a^* | 1 | 1 |
| *Varinicline* | 2 | - |
| **Antidepressants** | n | n |
| *SSRI* | 7 | 7 |
| *SNRI* | 2 | 2 |
| *Mirtazapine* | - | 2 |
| *Bupropion* | 2 | 2 |
| **Mood stabilizers** | n | n |
| *Lithium* | 7 | 2 |
| *Valproic acid* | 6 | 10 |
| *topiramate* | 4 | 1 |

1. Buprenorphine, methadone, long acting morphine= opioid agonist therapy (OAT)

**Supplementary Table 2**

Prescribed antipsychotics

| **N=87** | **39 (on clozapine)^1^** | **48 (other antipsychotic(s))^2^** |
| --- | --- | --- |
| **Oral medications** | n (mean daily dose, range) | n (mean daily dose, range) |
| *Amisulpride* | 3 (333.3 mg, 200-600) | 0 |
| *Aripiprazole* | 7 (9.3 mg, 5-15) | 7 (12.4 mg, 2-30) |
| *Haloperidol* | 0 | 1 (10 mg) |
| *Loxapine* | 1 (10 mg) | 3 (26.7 mg, 20-30) |
| *Lurasidone* | 0 | 1 (120 mg) |
| *Olanzapine* | 2 (17.5 mg, 10-25) | 17 (22.3 mg, 5-40) |
| *Paliperidone* | 1 (9 mg) | 1 (9 mg) |
| *Quetiapine* | 2 (350 mg, 300-400) | 5 (370 mg, 50-1050) |
| *Risperidone* | 0 | 1 (4 mg) |
| *Ziprasidone* | 0 | 1 (120 mg) |
| *Zuclopenthixol* | 2 (30 mg, 20-40) | 0 |
| **Long-acting injectables (LAIs)** | n (mean daily dose, range) | n (mean daily dose, range) |
| *Aripiprazole LAI* | 6 (15.1 mg, 14.3-19.0) | 13 (12.8 mg, 1.8-14.3) |
| *Flupentixol LAI* | 0 | 1 (3.6 mg) |
| *Haloperidol LAI* | 1 (3.6 mg) | 1 (7.1 mg) |
| *Paliperidone LAI* | 4 (5.4 mg, 3.6-7.1) | 12 (4.9 mg, 2.7-7.1) |
| *Risperidone LAI* | 0 | 1 (1.8 mg) |
| *Zuclopenthixol LAI* | 2 (21.4 mg, 17.9-25) | 4 (17.9 mg, 14.3-21.4) |

^1^: N=11 (28.2%) were on 1 antipsychotic (clozapine), N=25 (64.1%) were on 2 antipsychotics, and N=3 (7.7%) were on 3 antipsychotics

^2^: N=30 (62.5%) were on 1 antipsychotic, N=16 (33.3%) were on 2 antipsychotics, and N=2 (4.2%) were on 3 antipsychotics

**Supplementary table 3**

**Univariate association results for negative binomial regression**

| On clozapine | RR= 0.47, 95% CI= 0.28-0.80, p=0.005 |
| --- | --- |
| On antidepressant medications | RR=1.39, 95% CI=0.80-2.42, p=0.24 |
| On psychostimulant medication | RR=2.01, 95% CI=1.08-3.73, p=0.027 |
| Younger age (for every 10-years decrease) | RR=1.45, 95% CI=1.09-1.93, p=0.011 |
| On long acting injectable antipsychotics | RR=1.65, 95% CI=0.99-2.74, p=0.054 |
| Female Sex | RR=1.10, 95% CI=0.657-1.856, p=0.71 |
| On mood stabilizers | RR=1.98, 95% CI=0.71-2.02, p=0.51 |
| CPZ-equivalent daily dose | RR=0.937, 95% CI=0.86-1.03, p= 0.16 |
| ON SUD medications | RR=1.04, 95% CI=0.63-1.73, p=0.86 |
| On opioid agonist therapy | RR=0.93, 95% CI=0.55-1.57, p=0.79 |
